# Comment on: “Current and emerging strategies for myopia control: a narrative review of optical, pharmacological, behavioural, and adjunctive therapies”

**DOI:** 10.1038/s41433-025-04217-y

**Published:** 2026-01-17

**Authors:** Timothy J. Gawne, Andrew D. Pucker

**Affiliations:** 1 School of Optometry, University of Alabama at Birmingham, Birmingham, AL USA; 2 Eminent Ophthalmic Services, LLC, Milledgeville, GA USA

**Keywords:** Risk factors, Pathogenesis

## To the Editor:

We read with interest the review article by Maulvi et al. [1]. We found the review to be of high quality, but take issue with their citation of our paper [2]:“the 20-20-20 rule—looking at something 20 feet away for 20 s every 20 min—has been suggested as a simple method to reduce accommodative stress and visual fatigue.” First, both our paper and their review address myopia, not visual fatigue. Second, we point out that there is currently no human clinical data that the 20-20-20 rule will combat myopia, and there is considerable evidence from animal models that 20 s breaks from a myopiagenic activity (such as, presumably, reading and schoolwork) will be completely ineffective [2]. Anshel first proposed the 20-20-20 rule [3] and later noted that this was just a catchy way to remember to blink, breathe, and take breaks from near tasks [4]. It should also be noted that the 20-20-20 rule has been found to be ineffective for treating digital eye strain, which was the original intent of this treatment [5]. It has furthermore been proposed that a “20-20-2” rule—after 20 min of close work, children should gaze into the distance for at least 20 s, and they should also go outside for at least 2 h per day—would be more effective [6]. There is certainly strong evidence that having children spend time outside each day before becoming myopic would be useful in preventing myopia development [6, 7], but again, none whatsoever about a 20 s break every 20 min. Determining the optimal pattern and duration of “breaks” from myopiagenic activity should be a high priority, as it is possible that relatively modest changes in behavior could have powerful protective effects. However, until we know more, maybe something like “out and 2”—go outside and stay there for 2 h—would be a better approach.

## Commercial relationship disclosures

The authors have received research support from Abbvie Pharmaceuticals (ADP), Alcon Research, LLC (ADP), Bausch + Lomb (ADP), Meta (TJG), ORASIS Pharmaceuticals (ADP), and Topcon (ADP). The authors have served as consultants Bausch + Lomb (ADP), BRIM (ADP), and Lexitas Pharma Services (ADP). Dr. Pucker is an employee of Mintra Health and Electric Indigo, and he is the owner of Eminent Ophthalmic Services, LLC. Dr. Gawne holds several patents for optical anti-myopia treatments. None of these relationships have a direct impact on the submitted work.
